# Physical Exercise and Cognitive Function

**DOI:** 10.3390/ijerph19159564

**Published:** 2022-08-03

**Authors:** Diego Pastor, Juan Arturo Ballester-Ferrer, Laura Carbonell-Hernández, Sabina Baladzhaeva, Eduardo Cervello

**Affiliations:** Sports Research Centre, Department of Sport Sciences, Miguel Hernández University of Elche, 03202 Elche, Spain; juan.ballesterf@umh.es (J.A.B.-F.); laura.carbonell@goumh.umh.es (L.C.-H.); sabina.baladzhaeva@alu.umh.es (S.B.); ecervello@umh.es (E.C.)

## 1. Livelong Cognitive Function

Cognitive skills are relevant predictors of academic achievement, employability, socioeconomic success, health, and longevity [1]. It has been shown that cognitive skills are consolidated during adolescence and achieve maximum efficiency during youth [2]. However, during the aging process, there is a decrease in cognitive function with the years [3]. The cognitive function decline can be explained by the deterioration of the central nervous system during aging. Moreover, brain volume is reduced in regions including the frontal, parietal and temporal lobes and is possibly linked to the observed reductions in brain blood flow [4]. In addition, the decline in the hippocampus volume has been related to cognitive decline during aging [5]. Hippocampus is a cerebral structure that plays a central role in processes associated with declarative and visuospatial memory [6].

Moreover, some molecular changes can accelerate cognitive decline. These alternations include decreased levels of neurotrophic factors, such as BDNF (Brain-derived Neurotrophic Factor) and IGF-1 (Insulin-like Growth Factor-1), both leading to impaired neuronal survival and synaptic decline [7]. In addition, at a vascular level, reduced production of VEGF (Vascular Endothelial Growth Factor), a potent angiogenic factor, disrupts the creation of new blood vessels [8].

Cognitive decline affects activities of daily living in older people. However, it is essential to know that the decline rate is different between individuals and is influenced by many factors [1]. Consequently, it is crucial to identify the factors that can explain the slower rate of cognitive decline. For example, both physical activity and exercise have been shown to attenuate it [9]. Therefore, understanding the mechanisms responsible for this cognitive protection with physical exercise is essential.

## 2. Physical Inactivity, Physical Activity, and Physical Exercise Relationship with Cognitive Function

The study of physical inactivity’s effects on cognitive decline is an exciting way to understand the relevance of physical activity. Furthermore, physical inactivity has been considered the most significant health problem in the XXI century [10]. Physical inactivity leads to a range of adverse health consequences, including cognitive decline [11] and an increased risk of neurodegenerative diseases in older adults [12].

Physical activity and physical exercise are erroneously equated in some texts. It is important to differentiate both terms to understand the mechanisms regarding cognitive function [13]. Physical activity is any muscle movement that increases energy expenditure above the resting metabolic rate [14]. In contrast, physical exercise is a subset of physical activity that is planned, structured, repetitive and performed to improve or maintain one or more dimensions of fitness [14].

The term “physical exercise” is more common in the literature, as experimental research uses planned, structured and repetitive activities to study the relationship between physical exercise and cognition. A large body of research demonstrates the benefits of both acute [13,15,16,17,18,19] and chronic physical exercise [13,17,18,19,20,21] in improving cognitive function.

However, there are still many questions about the optimal type or dose of physical exercise to improve cognition efficiently. Moreover, we still do not know the influence of major moderators in this relationship. In the next section, we will discuss the role of some of the common moderators and highlight important questions that the research must still resolve to understand the relationship between physical exercise and cognitive function.

## 3. Physical Exercise and Cognitive Function. An Actual Point of View of Its Relationship

Many factors can moderate the physical exercise and cognitive response relationship [18,19]. Stillman, Cohen, Lehman and Erickson [18] classified the mechanisms moderating the exercise–cognition relationship in three levels. Thus, their classification explains cognitive improvements through (a) molecular and cellular modifications (level 1), (b) changes in brain structure and functional changes (level 2), and (c) behavioral and socioemotional changes (level 3). However, recent research has focused on the analysis of the first two levels of the exercise–cognition relationship; meanwhile, the behavioral influences remain unexplored [18]. As such, level one changes are most evident from the animal models, where observed benefits of physical exercise on the hippocampus imply enhanced spatial learning, memory, and exploration, all of which could be moderated by increased neurogenesis. [22]. In turn, the structural changes of level two have been reported in older adults, where physical exercise programs promoted increased hippocampal volume [23].

However, other moderators such as sleep, mood, or psychological well-being have been studied less extensively, despite some promising research [18]. For example, exercise was shown to improve psychological well-being [24] and modulate learning and academic achievement in young students [25,26]. Consequently, more research on level three should be interesting to improve our understanding of the exercise–cognition relationship.

Beyond these three levels, other moderators must be considered to increase the efficiency of the exercise–cognition relationship. We commonly use the acronym FITT to speak about distinct variables of the physical exercise prescription: Frequency, Intensity, Time and Type. There is much research on exercise intensity, which can significantly moderate the cognitive response after an acute exercise [27], with the most improvements attributable to high-intensity exercise. In terms of the time variable, there are some indications that low-volume sessions are conducive to the most benefits [27].

Regarding exercise type, most research articles analyze the effects of aerobic exercise on cognition. However, there is a growing research interest on the impact of resistance training on cognitive response, with some evidence that suggests positive improvements after both acute and chronic resistance exercise [17,20,28]. On the other hand, there is still a high heterogeneity in both the effects of resistance training and design protocols [20], and further research is needed on the dose–response effects for cognitive enhancement [17]. Moreover, we have an absolute lack of knowledge about frequency.

Individualized protocols should be considered to improve the efficiency of the exercise prescription for cognitive benefits. However, we still lack knowledge about specific populations. For example, most research has focused on older people and children [19], but adolescents and young people have been studied less [19]. Moreover, we already know that sex can be a powerful moderator in the exercise–cognition relationship [21,29], but there are questions remaining about the influence of sex. For example, some studies have observed higher levels of BDNF in men than in women, both after acute and chronic physical exercise [30]. In contrast, other studies have found better cognitive response after aerobic exercise in women rather than in men [29].

In conclusion, future research should aim at resolving these questions to promote evidence-based exercise prescription to improve cognitive function.
